# Natural Statistics as Inference Principles of Auditory Tuning in Biological and Artificial Midbrain Networks

**DOI:** 10.1523/ENEURO.0525-20.2021

**Published:** 2021-06-04

**Authors:** Sangwook Park, Angeles Salles, Kathryne Allen, Cynthia F. Moss, Mounya Elhilali

**Affiliations:** 1 Department of Electrical and Computer Engineering, Johns Hopkins University, Baltimore, MD, 21218; 2 Department of Psychological and Brain Sciences, Johns Hopkins University, Baltimore, MD, 21218

**Keywords:** big brown bat, biomimetic network, IC, machine learning, spectro-temporal receptive fields

## Abstract

Bats provide a powerful mammalian model to explore the neural representation of complex sounds, as they rely on hearing to survive in their environment. The inferior colliculus (IC) is a central hub of the auditory system that receives converging projections from the ascending pathway and descending inputs from auditory cortex. In this work, we build an artificial neural network to replicate auditory characteristics in IC neurons of the big brown bat. We first test the hypothesis that spectro-temporal tuning of IC neurons is optimized to represent the natural statistics of conspecific vocalizations. We estimate spectro-temporal receptive fields (STRFs) of IC neurons and compare tuning characteristics to statistics of bat calls. The results indicate that the FM tuning of IC neurons is matched with the statistics. Then, we investigate this hypothesis on the network optimized to represent natural sound statistics and to compare its output with biological responses. We also estimate biomimetic STRFs from the artificial network and correlate their characteristics to those of biological neurons. Tuning properties of both biological and artificial neurons reveal strong agreement along both spectral and temporal dimensions, and suggest the presence of nonlinearity, sparsity, and complexity constraints that underlie the neural representation in the auditory midbrain. Additionally, the artificial neurons replicate IC neural activities in discrimination of social calls, and provide simulated results for a noise robust discrimination. In this way, the biomimetic network allows us to infer the neural mechanisms by which the bat’s IC processes natural sounds used to construct the auditory scene.

## Significance Statement

Recent advances in machine learning have led to powerful mathematical mappings of complex data. Applied to brain structures, artificial neural networks can be configured to explore principles underlying neural encoding of complex stimuli. Bats use a rich repertoire of calls to communicate and navigate their world, and the statistics underlying the calls appear to align with tuning selectivity of neurons. We show that artificial neural network with a nonlinear, sparse and deep architecture trained on the statistics of bat communication and echolocation (Echo) calls results in a close match to neurons from bat’s inferior colliculus (IC). This tuning optimized to yield an effective representation of spectro-temporal statistics of bat calls appears to underlie strong selectivity and noise invariance in the IC.

## Introduction

Biological neural circuits are believed to provide an efficient code of the sensory world, which allow us to process complex and dynamic stimulus information from our surroundings. Perception of an auditory scene is created by neural activity filtered through several stages of feed-forward and feedback sensory processing. Sound pressure of an acoustic signal is first transduced into a bio-electrical signal in the cochlea. Subsequently, the bio-electrical signal is relayed through the auditory pathway. The inferior colliculus (IC) is an auditory hub that receives ascending inputs from brainstem nuclei and sends information through the thalamus to the auditory cortex, while it also receives descending inputs from auditory cortex (Casseday et al., 2002). The IC encodes complex auditory features such as frequency sweep rate (Williams and Fuzessery, 2010) and patterning (Gordon and O’Neill, 1998) that are necessary for identification of complex auditory objects and therefore plays a key role in representing these objects in a natural listening environment.

Echolocating bats build a representation of their surroundings by emitting ultrasonic vocalizations and processing the features of returning echoes to compute the location and features of targets and obstacles in the environment. Bats must rapidly process sonar echoes while concurrently parsing environmental noise and calls emitted by conspecifics. In this complex and rapidly changing auditory scene, the bat’s brain efficiently encodes acoustic stimuli and allows the animal to accurately track prey, avoid obstacles, and communicate with conspecifics while dynamically navigating a 3D environment. Humans and other animals face similar challenges in the course of their natural acoustic behaviors. With the goal of elucidating principles underlying auditory scene analysis in the midbrain, we examine the relationship between statistics of the rich acoustic repertoire of bat calls and neural response patterns in the bat’s IC to explore artificial networks tuned to map natural statistics in these calls and identify emergent properties that match responses in the IC.

Here, we test the hypothesis that the bat’s auditory midbrain is optimized to accurately represent the natural statistics in the sounds and echoes that exist in the bat’s environment [particularly social and echolocation (Echo) calls]. Past research has suggested that the IC plays a major role in the representation and mapping of communication sounds that give rise to specialized encoding of natural sounds along the ascending auditory system (Aitkin et al., 1994; Suta et al., 2003). An earlier study in the Mexican free tailed bat suggested a possible correspondence between tuning characteristics of individual IC neurons and properties of natural calls from conspecific sounds (Brimijoin and O’Neill, 2005; Andoni et al., 2007). In the current study, we corroborate this relationship in a different species and further probe constraints and implications of such optimal encoding of natural sounds on auditory signal processing in a complex scene.

We recorded vocalizations from socially housed bats and analyzed the spectro-temporal statistics of natural sounds [e.g., frequency modulation (FM) velocity, directionality]. Using the database of collected statistics, we built an artificial network, which projects sounds onto a latent space that efficiently represents statistics of these natural sounds in a strategy of signal reconstruction (Smith and Lewicki, 2006). This computational model offers a biomimetic architecture whose main operation is to capture the statistics of natural bat calls, without information about the function of biological neurons. We then ask: Does the emergent tuning of this artificial network match properties of biological neurons in the big brown bat IC? To answer this question, we also recorded responses to sound stimuli, spectro-temporal ripples, from individual neurons in the IC of big brown bats.

**Figure 1.**
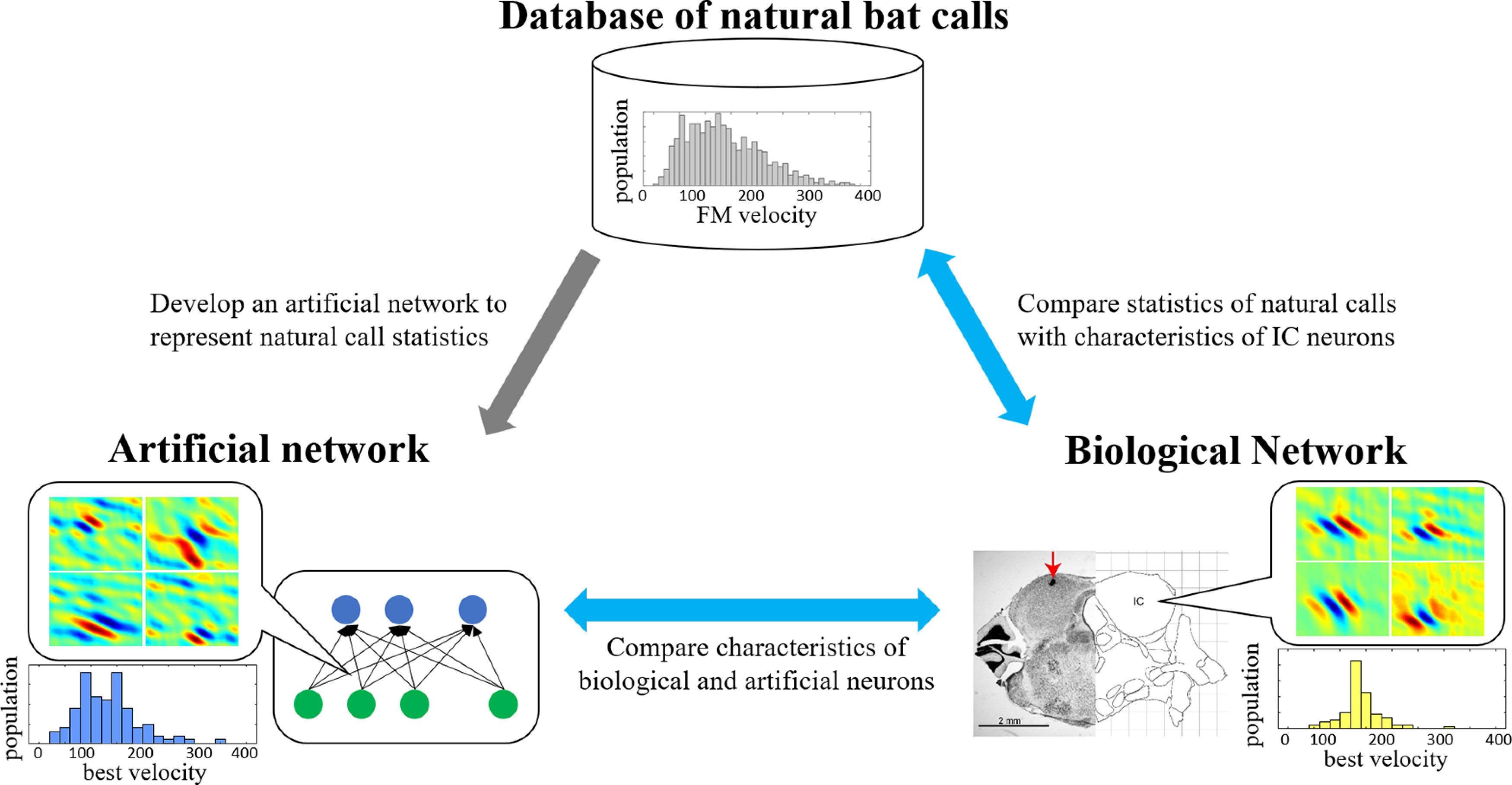
Overview of study foci. A database of natural calls from a colony of big brown bats is collected and analyzed for its auditory characteristics. Shown in the figure is a distribution of FM velocities. Right, Tuning characteristics of biological neurons from the big brown bat IC are derived using STRF method, and properties of biological neurons are derived (e.g., BV). Shown in the figure is a brain slice identifying the location of the IC in the big brown bat (from Salles et al., 2020). Left, Computational models with various configurations are examined and emergent tuning properties of artificial networks are derived to compare against statistics of natural calls as well as biological neurons.

It is known that the spectro-temporal receptive fields (STRFs) suggest a reasonable linear-approximation of neural responses as a transfer function from acoustic stimuli and those are usually used to explore auditory characteristics of the neurons (Depireux et al., 2001; Andoni et al., 2007; Elhilali et al., 2013). We extracted STRFs from IC and artificial neurons and calculated auditory characteristics from these neural response functions (Kowalski et al., 1996; Poon and Yu, 2000). The spectro-temporal tuning characteristics of biological neurons were then compared with both the statistics of natural calls as well as emergent tuning of artificial neurons. By varying the configuration of the artificial network, we employed the theoretical network as springboard to examine possible constraints on the configuration of midbrain networks, and gauge the validity of the hypothesis linking biological encoding in the mammalian midbrain to efficient representation of natural sound statistics. While various artificial neural networks can be optimized to reconstruct an input sound from compressed feature on latent space, finding an architecture that closely emulates the biological network provides insights into the underlying functional role of certain brain nuclei. Here, we examine the relationship between the optimal encoding of natural statistics in bat calls and its role in facilitating robust selectivity across sound classes in the repertoire. The graphical abstract in Figure 1 shows an overview of the approach taken in this work.

## Materials and Methods

### Collection of bat’s vocalization

#### Animals

Big brown bats (*Eptesicus fuscus*) were collected from an exclusion site under a state permit. All experimental procedures were conducted in accordance with a protocol approved by an Institutional Animal Care and Use Committee. A total of ∼100 bats were housed in our laboratory and used for vocal data recordings, and four (two male, two female) bats were used for neurophysiological data collection.

#### Audio recordings for training the biomimetic network

A bat call library was built from audio recordings of bats housed in a vivarium room where the temperature is kept at 70–80°F, and humidity is kept at 30–70%. This room holds ∼100 bats in groups of one to six separated in mesh cages. The recordings were made for 2 d using an Avisoft CM16/CMPA ultrasonic microphone and the Avisoft-RECORDER software. Mono audio was recorded at a sampling rate of 300 kHz.

Natural call recordings from big brown bats were processed to extract meaningful segments. An energy-based signal activity detection was performed on the entire database to remove the silences between calls and to split the recordings into segments containing bat calls (Park et al., 2014). As a result, we constructed species-specific databases containing 17,713 calls (∼10 min) for big brown bats. This call database was used for training artificial networks. The data were divided into a training set (15,000 randomly selected calls) to learn network parameters and test set (remaining 2713 calls) for verifying the network.

#### Social calls for natural sound representation

**Figure 2.**
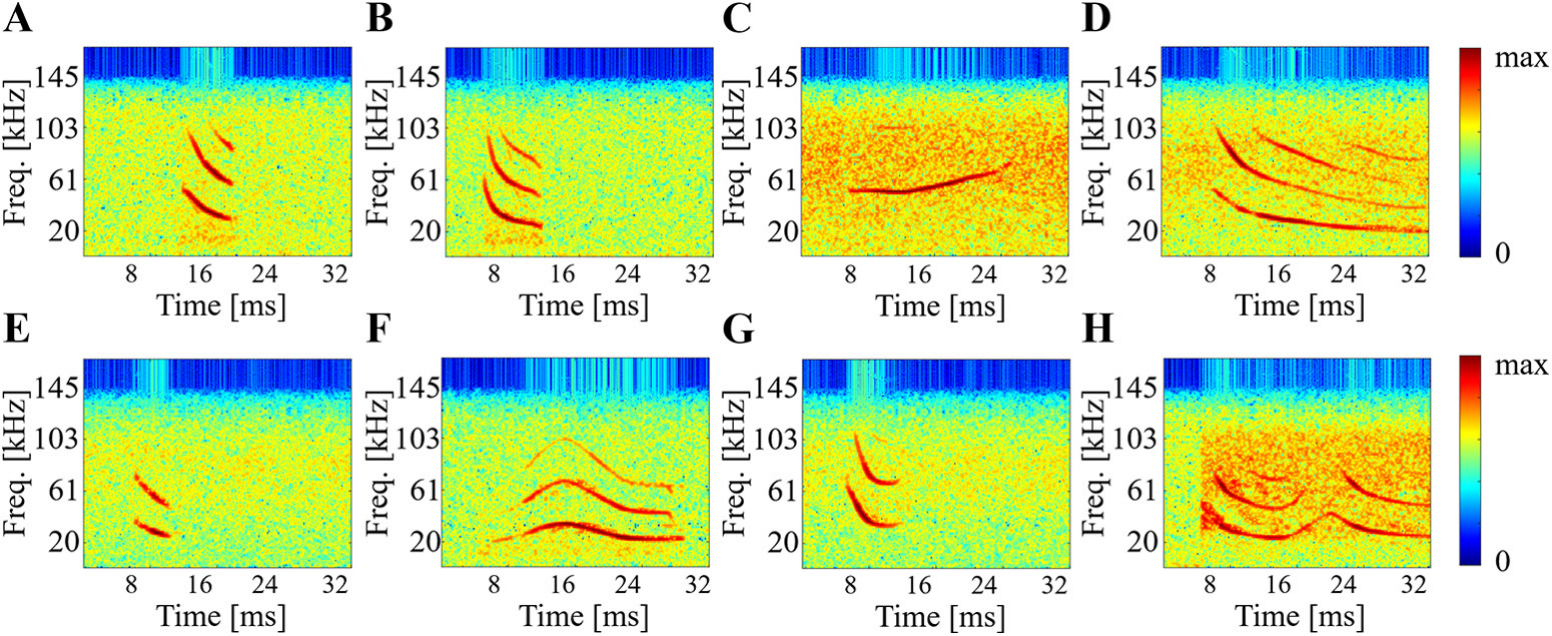
Example spectrograms of eight bat calls in social call database. ***A***, Echo. ***B***, FMB. ***C***, UFM. ***D***, LFM. ***E***, SFM. ***F***, CS. ***G***, Hook. ***H***, Long-wave.

To investigate discriminability in the artificial network, we used a social call database that includes 26 audio clips for eight different types of bat calls (Fig. 2). These types include six calls, as defined in (Wright et al., 2013), specifically, Echo, frequency-modulated bout (FMB), upward frequency modulated (UFM), long frequency modulated (LFM), short frequency modulated (SFM), and chevron-shaped (CS); in addition to two additional calls types, long-wave and hook, which resemble a hook in time-frequency space. All audio clips were up-sampled from 250 to 300 kHz.

### Neurophysiological IC data

Recordings of neural responses from IC neurons were used to perform two separate analyses: (1) characterize receptive field tuning of IC neurons; and (2) examine discriminability of IC neurons to different conspecific calls. Methods for receptive field analysis are described below in Analysis of neuronal responses, while data used for discriminability analysis are described below in Neural discriminability of conspecific calls.

#### Receptive field recordings

A head-post was adhered to the skull of bats for head fixation as described previously (Macías et al., 2018). The IC was located using skull and brain landmarks and a surgical drill was used to make a ≤1-mm diameter craniotomy preserving dura. The neurophysiological recordings were performed in a sound-attenuating and electrically shielded chamber (Industrial Acoustics Company). Each bat was restrained individually in a custom-made foam mold and the head was fixed by the head-post. Recording sessions were conducted over three to five consecutive days, each one lasting no more than 4 h. Water was offered to the bats every 2 h. No drugs were administered during recordings. During recordings a silver wire for grounding was placed in between muscle and skull ∼5 mm rostral to the craniotomy site. The 16-channel recording probe (Neuronexus A1x16-5 mm-50-177-A16) was inserted into the brain using a micromanipulator. The surface of the brain was registered as 0 μm for depth reference and the probe was advanced in 10 μm steps using a hydraulic microdrive (Stoelting Co). Recordings were taken at least 100 μm apart. An OmniPlex D. Neural Data Acquisition System recording system (Plexon) was used to obtain neural responses with 16-bit precision and 40-kHz sampling rate. A transistor-transistor-logic (TTL) pulse for each stimulus presentation was generated with the National Instrument card used for stimulus presentation and was recorded on channel 17 of the analog channels of the acquisition system for synchronization of acoustic stimuli and neural recordings. The stimuli were recorded on channel 18 of the acquisition system to corroborate synchronization.

#### Moving ripple stimuli

A set of ripple stimuli was generated to estimate STRFs of IC neurons (Kowalski et al., 1996; Depireux et al., 2001; Andoni et al., 2007). Ripples are modulated noise stimuli that are dynamic both in time and frequency. Each ripple can be described as

**Figure 3.**
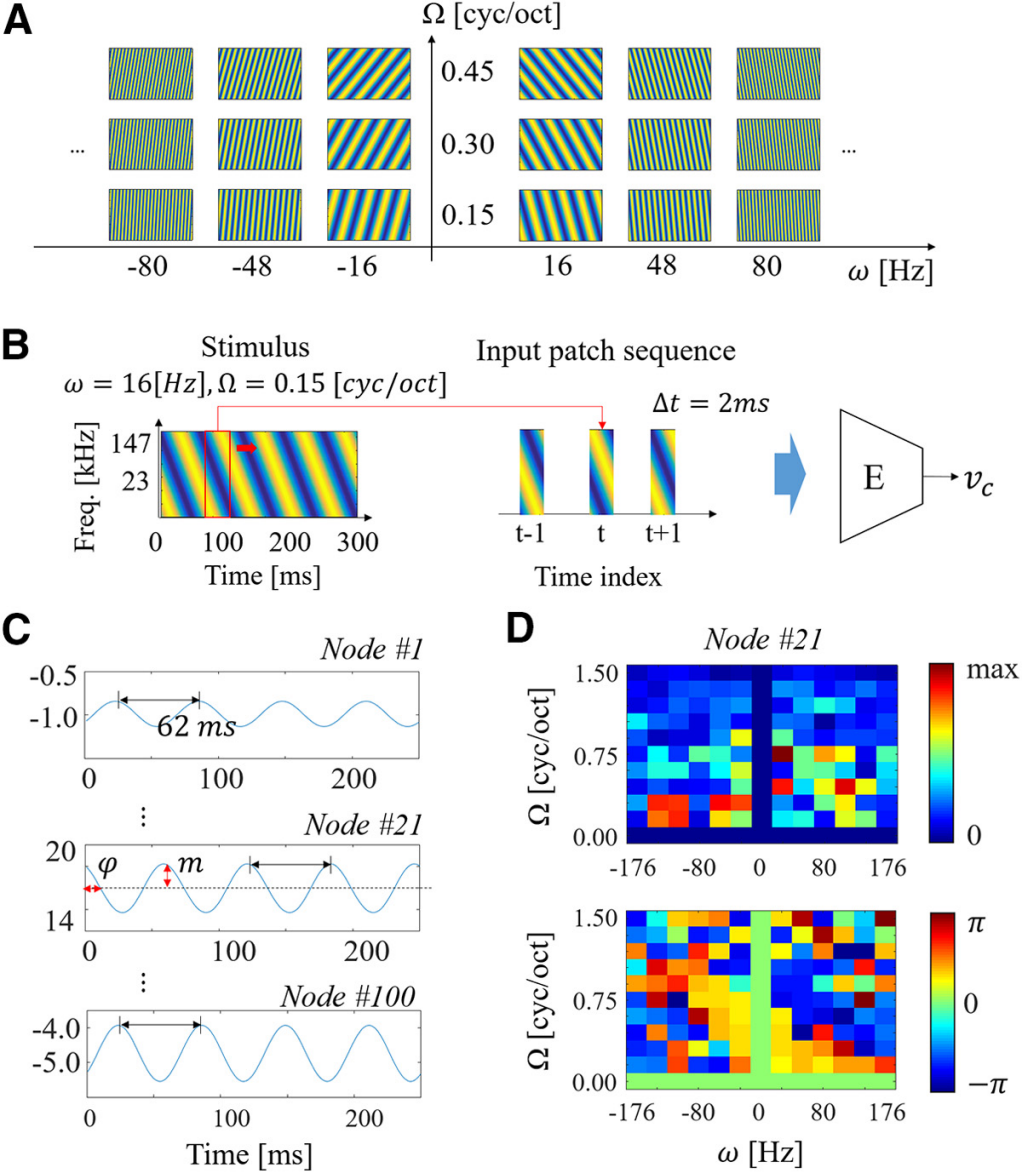
RTF extraction. ***A***, A subset of ripple stimuli. ***B***, Input patch sequence configuration from a ripple stimulus to a code vector for characterizing the network. ***C***, Examples of responses on each node (each element of the code vector), and definition of magnitude *m* and phase *ϕ* in a ripple response. ***D***, Magnitude and phase plots on one of nodes.

(1) S(t,x)=1 + ΔA×sin(2π(ωt + Ωx) + ϕ),

where *t* and *x* are indices for time and octave scaled frequency. ΔA and *ϕ* are amplitude and a phase, respectively. And *ω* and Ω represent modulation rates along temporal (Hz) and spectral (cyc/oct) axes. The temporal and spectral modulation parameters were varied from −176 to 176 Hz in steps of 32 Hz and 0.0–1.5 cyc/oct in steps of 0.15 cyc/oct spectrally (Fig. 3*A*). Each ripple spanned 6.66 octaves from 1.2 to 121 kHz and was 300 ms in duration.

#### Audio playbacks for neural recordings

Extracellular recordings from the IC of awake animals were taken while they passively listened to broadcast of either ripple stimuli, or pure tones at 70 dB. All stimuli were generated at a sampling rate of 250 kHz using a National Instruments card (PXIe 6358) and transmitted with a calibrated custom-made electrostatic ultrasonic loudspeaker connected to an audio amplifier (Krohn-Hite 7500). The loudspeaker was placed at 60 cm (for all ripple and pure tones stimuli) from the bat’s ear. The frequency response of the loudspeaker was compensated by digitally filtering the playback stimuli with the inverse impulse response of the system as described previouosly (Luo and Moss, 2017).

Frequency tuning curves were built by recording neural responses to pure tones of 5-ms duration (with 0.5-ms ramping rise and fall). The tones ranged between 20 and 90 kHz (in 5 kHz steps) and the sound pressure levels ranged from 20 to 70 SPL (10 dB steps). At each recording site first, we played 20 repetitions of the randomized ripple stimulus and then 15 repetitions of each of the randomized pure tones at a different SPL.

#### Analysis of neuronal responses

For the analysis of auditory tuning in response to ripple and pure tone stimuli, responses were sorted offline, then single units were detected using the program Wave_clus (Quiroga et al., 2004). Each individual waveform was inspected and the acceptance threshold for clusters was <10% of spikes with <3-ms interspike interval, consistent with the neuronal refractory period. Any sites that showed no response to ripple stimuli were excluded from the spike sorting and further analysis in line with procedures used in other studies (Poon and Yu, 2000; Escabi and Schreiner, 2002; Andoni et al., 2007). After spike sorting, the Euclidian distance error between the mean and variance of number of spikes across trials was computed. Units whose error is <1.0 were selected for further analysis, following a Poisson model of spike representation (Corrado et al., 2005; Schwartz et al., 2006). This analysis resulted in 108 single units used for the current study.

##### Neurophysiological STRFs

At each recording site, ripple stimuli were repeated 10–20 times in a randomized order for each repetition. A PST histogram was calculated from the spike time sequence of each ripple; then histograms were folded into 32-point periods. The strength and phase of the response to each ripple were estimated directly from the fundamental component obtained by applying a 32-point fast Fourier transform (FFT) to the period histogram. Magnitude and phase responses to each ripple were combined together into a magnitude matrix M(Ω,ω) and a phase matrix Φ(Ω,ω), respectively. To derive a ripple transfer function (RTF), which is a representation of a STRF in the modulation domain, M(Ω,ω) and *Φ*(Ω, ω) were expanded to four quadrants in the modulation domain spanning from −176 Hz and −1.5 cyc/oct to 176 Hz and 1.5 cyc/oct as $M_{e}\left(\text{Ω},\omega \right)=M_{e}^{*}\left(-\text{Ω},-\omega \right)=M(\text{Ω},\omega )$ and $\Phi_{e}\left(\text{Ω},\omega \right)=\Phi_{e}^{*}\left(-\text{Ω},-\omega \right)=\Phi (\text{Ω},\omega )$ based on a symmetric property around the origin (Depireux et al., 2001; Andoni et al., 2007). As a result, the RTF was formulated as

(2) $$T\left(\text{Ω},\omega \right)=M_{e}\left(\text{Ω},\omega \right)e^{j\Phi_{e}\left(\text{Ω},\omega \right)},$$

where $j=\sqrt{-1}$. Finally, a STRF was obtained by performing 2D inverse FFT on the RTF as

(3) $$STRF\left(x,t\right)=F_{t,-x}^{-1}[T(\text{Ω},\omega )],$$

where $F^{-1}$ designates the 2D inverse FFT along each axis in the modulation domain.

#### Neural discriminability of conspecific calls

In order to examine selectivity of IC neurons to calls from the bat’s natural repertoire, we re-used neural data previously collected in an earlier study (Salles et al., 2020), where we collected neuronal responses to Echo calls versus FMB social calls. The study followed the same methodology for data collection as described here. Wave_clus was used to detect and classify single units from the recordings. The spikes responding to either FMB or Echo were counted in windows of 25 ms in duration, starting 5 ms after stimulus onset. Some units with an average of less than five spikes over 20 times recordings were excluded because they were considered as a non-responsive unit to the stimulus. Multiunit activity was determined from interspike intervals with <3 ms that were inconsistent with neuronal refractory period; and units with >10% of spikes with <3-ms interspike interval were excluded from analysis. As a result, total 575 units were finally obtained and their responses are used in the present work to contrast neural discriminability between Echo and FMB calls with artificial neurons.

### Responses in artificial neurons

#### Artificial network front-end processing

To develop a biomimetic architecture, a biologically-inspired auditory spectrogram is used as input for the network (Shamma, 1985a,b; Yang et al., 1992; Wang and Shamma, 1994). The auditory spectrogram incorporates four processing stages that emulate peripheral processing in the mammalian system: cochlear filtering, auditory-nerve transduction, hair cell responses, and lateral inhibition (Chi et al., 2005). Briefly, an incoming acoustic waveform is analyzed along a bank of constant-Q filters spanning a logarithmic scale. Then, each frequency channel undergoes a high-pass, nonlinear compression and low-pass filtering followed by lateral inhibition across frequency, following the implementation available in the NSL toolbox (Chi and Shamma, 2005) with the following settings: the frame length was set to 0.2 ms without overlap, and each octave was represented with 24 channels (i.e., 128 channels over 5.33 octaves). Octave-scaled center frequencies were represented as $f_{c}=440\times 2^{\left((c-32)/24+\gamma \right)}$ where $f_{c}$ is a center frequency of the $c^{th}$ channel, and *γ* is a constant factor of octave shift (γ=4.38). Inputs to the artificial network were sampled as square patches of the spectrogram spanning 128 frequency channels (i.e., 5.33 octaves) and 160 time samples (i.e., 32 ms).

#### Structure of artificial network

An artificial neuron, i.e., node mimicking a biological neuron, is mathematically modelled by a linear combination of prenode outputs and a nonlinear activation function. An artificial network is constructed by connecting a large number of nodes to each other. Using nonlinear activation functions enables the network to perform nonlinear computations on feedforward propagation. For this study, we favored a generative architecture using an autoencoder composed of an encoder, which compresses original data into a compact code, and a decoder, which reconstructs the original signal from that code (Baldi, 2012; Doersch, 2016). The intuition is to directly test our hypothesis that the network would infer a statistical model of the training dataset of natural calls, and if successful should allow a faithful reconstruction of the inputs.

**Figure 4.**
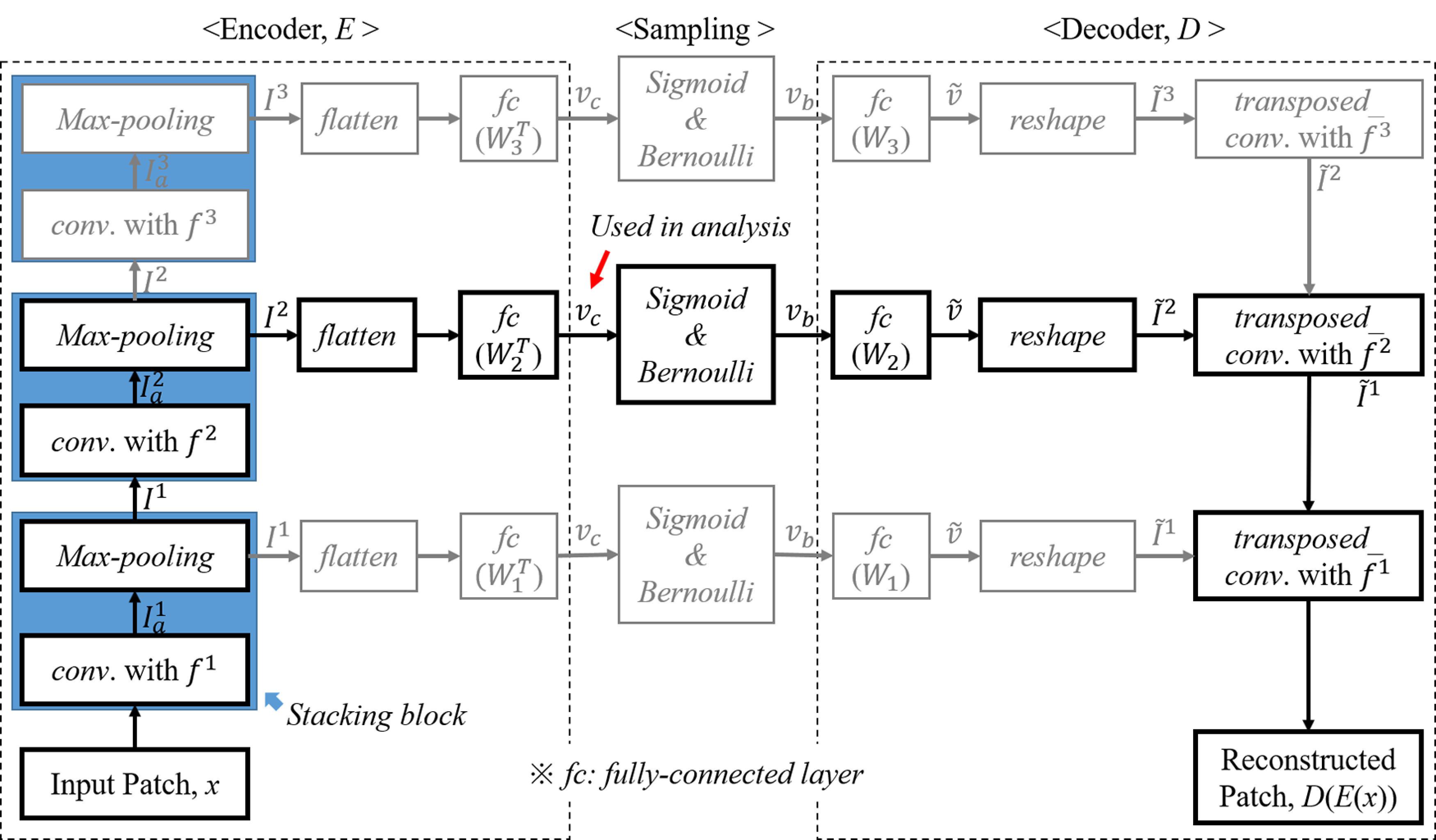
Convolutional layered autoencoder structure for biomimetic network. The flow denoted in black shows a double stacking structure as a standard example. Based on this structure, a deeper structure can be constructed by stacking more modules, on the other hand, a shallow structure is created by removing a module on the top of the standard example.

The proposed architecture is shown in Figure 4. First an encoder stage ***E*** is composed of convolutional layers, pooling layers, and a fully connected layer. A latent vector represents compressed features learned from the input data. A decoder stage ***D*** composed of reverse operations using transposed convolutions, reconstructs the input features from a latent vector. A sampling stage, interposed between the encoder and decoder, emulates neural activity yielding sparse binary activations.

Using the same general building block composed of convolution and pooling layers, this study investigates various configurations of the network by varying (1) depth, which is the number of blocks (in Fig. 4, the black-flow shows a double stacking structure as an example); a deeper network can be constructed by stacking more blocks, on the other hand, a shallow network can be created by removing a block; (2) nonlinearity, by varying the slope of nonlinear activation function employed; and (3) sparsity, by controlling the density of sampling in the latent space.

**Figure 5.**
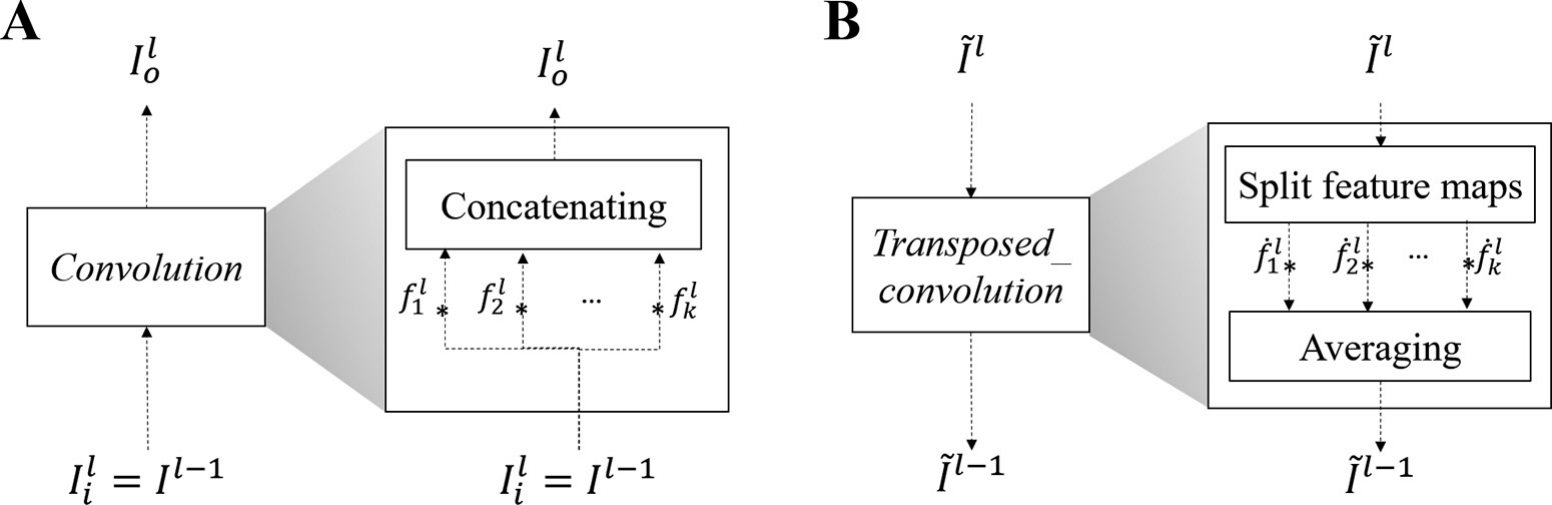
Operations using multiscale filters. ***A***, Convolution using multiscale filters. ***B***, Transposed convolution using multiscale filters.

The encoder architecture ***E*** follows a convolutional neural network (CNN) framework to reduce the number of trainable parameters, hence controlling for overfitting issues and generalizability to unseen data (Dietterich, 1995). The convolutional layers compute output feature maps using 2D convolutions between input feature maps and several filters as follows:

(4) $$I_{o}^{l}\left[f,t,k\right]=\underset{\xi ,\tau ,m}{\sum }I_{i}^{l}\left[\xi ,\tau ,m\right]f^{l}[\xi -f,\tau -t,m,k],$$

where f,t,l,k and *m* are indices for spectral, temporal, layer, channel of output feature map, and channel of input feature map, respectively. $I_{i}$, $I_{o}$, and $f^{l}$ are feature maps for input and output, and convolutional filter applied in the $l^{th}$ layer, respectively. Multiscale filters are employed in each convolutional layer to balance broad span (in time and frequency) versus localized analyses. Then, output feature maps concatenate filter outputs using multisized filters (Fig. 5*A*; Szegedy et al., 2015). Specifics of both filter composition and dimensions of intermediate feature maps are summarized in Table 1. Neural activation by an acoustic feature is emulated by applying a nonlinear function after convolution as follows:

(5) $$I_{a}^{l}\left[f,t,k\right]=\text{max}(I_{o}^{l}\left[f,t,k\right],\alpha \times I_{o}^{l}\left[f,t,k\right]),$$

where *α* is a constant within an interval [0, 1] (Maas et al., 2013). Next, pooling layers compress the output from the previous convolutional layer by extracting a maximum among some values enclosed by a non-overlapping window (i.e., max pooling) $I^{l}$ (Scherer et al., 2010). As a result, the width and height of the output are reduced by half. At the top of the encoder, a fully connected layer is applied for mapping into a latent space, which involves natural statistics requiring to reconstruct original input, as $v_{c}=W_{l}^{T}\times flatten(I^{L})$ where $I^{L}$ is a feature map in the last pooling layer, *W* is weight matrix in the fully connected layer, and flatten(.) is a reshape function from a 3D tensor to a vector.

**Table 1.** Description of network parameters, midlevel feature maps, and input.

Category	Description	Parameter set
Multiscale convolution filters	For the 1st convolution layer	$f^{1}=\left[f_{1}^{1},f_{2}^{1},f_{3}^{1},f_{4}^{1}\right],where$ $f_{1}^{1}\in R^{3\times 3\times 1\times 2},f_{2}^{1}\in R^{5\times 5\times 1\times 2},f_{3}^{1}\in R^{7\times 7\times 1\times 2},f_{4}^{1}\in R^{9\times 9\times 1\times 2}$
		
	For the 2nd convolution layer	$f^{2}=\left[f_{1}^{2},f_{2}^{2},f_{3}^{2}\right],where$ $f_{1}^{2}\in R^{3\times 3\times 8\times 4},f_{2}^{2}\in R^{5\times 5\times 8\times 4},f_{3}^{2}\in R^{7\times 7\times 8\times 4}$
		
	For the 3rd convolution layer	$f^{3}=\left[f_{1}^{3},f_{2}^{3}\right],where$ $f_{1}^{3}\in R^{3\times 3\times 12\times 8},f_{2}^{3}\in R^{5\times 5\times 12\times 8}$
		
Weight matrix	For the fully connected layer	$W_{1}\in R^{40960\times 100}$, $W_{2}\in R^{15360\times 100}$, $W_{3}\in R^{5120\times 100}$
		
Input patch	Network input	$x\in R^{128\times 160\times 1}$
Feature maps	In encoder	$I^{1}\in R^{64\times 80\times 8},I^{2}\in R^{32\times 40\times 12},I^{3}\in R^{16\times 20\times 16}$
	In decoder	$I^{1}\in R^{64\times 80\times 8},I^{2}\in R^{32\times 40\times 12},I^{3}\in R^{16\times 20\times 16}$

In the middle stage, a binary code vector $v_{b}$ is generated by performing a Bernoulli sampling process. A sigmoid function is applied to the latent vector to calculate prior probabilities. Thus, the output of the middle stage is represented as $v_{b}=Bernoulli(\sigma (v_{c}))$ where σ(.) is a sigmoid function.

The decoder ***D*** is composed of a fully connected layer and transposed convolution layers. In the fully connected layer, a latent vector is expanded into an initial space as $\hat{v}=W_{l}\times v_{b}$, and the vector $\hat{v}$ is reshaped to a 3D tensor as a set of initial feature maps as ${\hat{I}}^{l}=reshape(\hat{v})$. From initial feature maps, a transposed convolution using multiscale filters is sequentially performed until the output has the same dimensions as the input patch (Radford et al., 2015; Shelhamer et al., 2017). Convolutional filters used in the encoder are applied for transposed convolution after transposing input channel from output channel dimension as ${\dot{f}}^{l}[f,t,k,m]$. A transposed convolution using multiscale filters is performed in three steps (Fig. 5*B*). First, the input feature map ${\hat{I}}^{l}$ is split into submaps, $\left[{\hat{I}}_{1}^{l},{\hat{I}}_{2}^{l},...,{\hat{I}}_{N}^{l}\right]$, as many as the number of filters. Second, transposed convolution is individually performed for each pair of submap and filter. Finally, a set of output feature maps is obtained by averaging the results of the second step.

#### Training artificial network

The network was trained using the cost function:

(6) $$L=\frac{1}{2}\underset{n}{\sum }[{\left(x_{n}-D(E\left(x_{n}\right))\right)}^{2}+\lambda {\left(\rho -\underset{i}{\sum }\sigma (v_{c_{i}})\right)}^{2}],$$

where $x_{n}$ is an input patch with respect to the $n^{th}$ index, E(.) represents an encoder function while D(.) is for a decoder, and *ρ* means the average number of active nodes. The first term represents the mean square error between an input patch and its reconstruction by the autoencoder. The sparse constraint prevents overfitting as well as emulates sparsity of active neurons in the brain. Let *Y* be a random variable representing the number of active nodes by the Bernoulli process. Then, the distribution known as the Poisson binomial distribution is denoted as

(7) $$\mathrm{Pr}\left(Y=\rho \right)=\underset{A}{\sum }[\underset{i\in A}{\prod }\sigma (v_{c_{i}})\underset{j\in A^{c}}{\prod }(1-\sigma \left(v_{c_{j}}\right))],$$

where *A* is a set whose elements are possible combination for choosing *ρ* nodes from *N* nodes. This distribution can be approximated by Binomial(N,μ/N) where $\mu =\underset{i}{\sum }\sigma (v_{c_{i}})$(Choi and Xia, 2002). The network training was implemented using TensorFlow (Abadi et al., 2016). AdamOptimization was applied for an optimizer with 1.0e−4 learning rate. And, *λ* was set to 1.0e−4. For more details, readers can find the implementation on http://www.github.com/JHU-LCAP/BioSonar-IC-model/.

Comparisons between the statistic of bat calls and artificial neurons were performed to infer the network configuration that best matches the characteristics of IC neurons (as explained next). The best configuration composed of a triple stacking network, a parameter of nonlinearity α=0.2 in Equation 5 and 10% sparsity constraint in Equation 6.

#### Biomimetic STRFs

**Figure 6.**
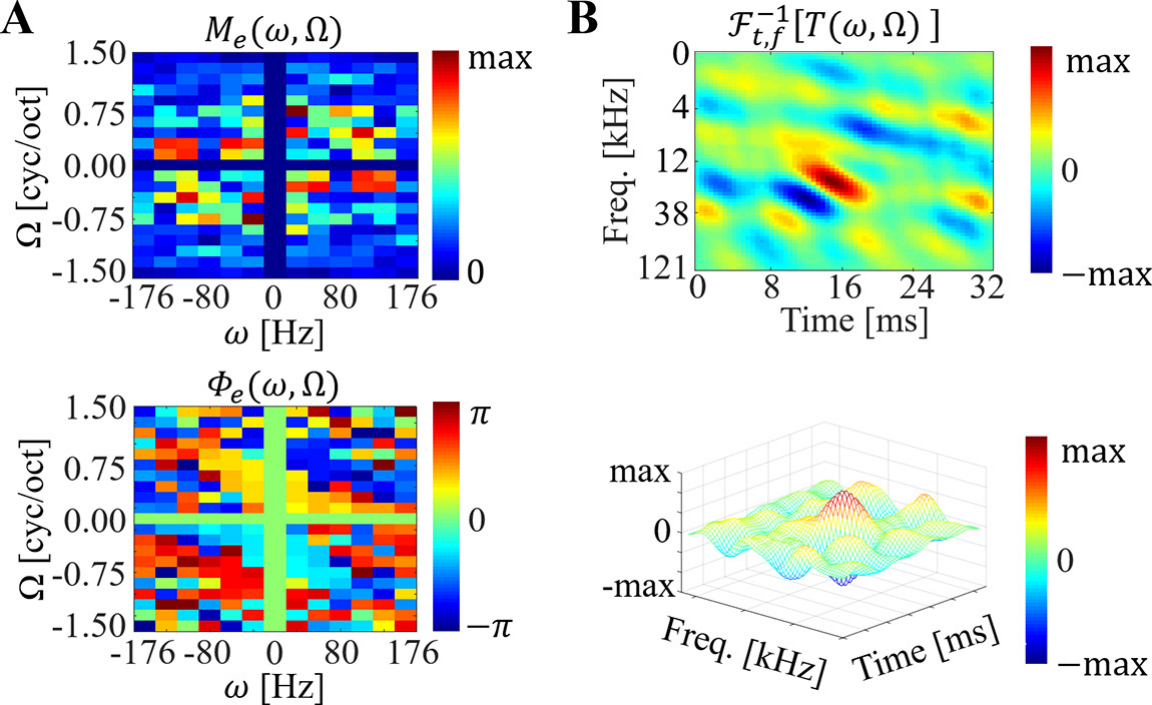
STRF calculation. ***A***, expanded magnitude and phase matrices which are matching to Figure 2*D*. ***B***, 2D (above) and 3D (bottom) representation of the STRF that is obtained by performing 64 × 64 interpolation and Gaussian smoothing sequentially. In 2D representation, red area represents excitation regions while blue represents inhibition regions.

Once trained, the network was interrogated following the same procedure as biological neurons. The same ripple stimuli were given as input to the network and activity of the nodes before applying the sigmoid activation and the Bernoulli sampling, $v_{c}$ in Figure 4 was characterized. Each ripple was transformed into an auditory spectrogram (as described earlier). A sequence of input patches for each ripple were then composed by applying a sliding window (window length: 160 frames) in every 2 ms (sliding step: 10 frames; Fig. 3*B*). Input patches in the sequence were consecutively fed into the pretrained encoder, then a latent vector $v_{c}$ was obtained every 2 ms. The same procedure for extracting biological STRFs was followed (see above, Analysis of neuronal responses). To find the magnitude *m* and phase *ϕ* of the responses, we performed a 32-point FFT and derived the magnitude and unwrapped phase of the fundamental component (Fig. 3*C*). By repeating this procedure for all ripples, the magnitude and phase were collected in a matrix M(Ω,ω) and a Φ(Ω,ω), respectively (Fig. 3*D*). These modulation responses were then converted into time-frequency STRF profiles by performing a 2D inverse FFT on the RTF (Fig. 6). Note that, in this study, all network architectures employed a total 100 artificial neurons (spanning a 100D latent space) so that 100-biomimetic STRFs were used for analysis.

### Analysis of auditory characteristics

#### Natural statistics and Auditory characteristics

##### FM velocity (statistics of bat calls)

To characterize conspecific vocalizations, we calculated FM velocities of each call segment in our database. Since moving ripples were used as bases components of the Fourier modulation domain (Singh and Theunissen, 2003), we derived auditory spectrograms of each call, then performed a 2D FFT after mean subtraction to remove constant components. $T_{c}\left(\text{Ω},\omega \right)=F_{f,t}[S\left(f,t\right)-\bar{S}]$ where $F_{f,t}[.]$ is the 2D FFT, *S* is an auditory spectrogram of a bat call, and $\bar{S}$ is its mean over the time and frequency axes. A velocity line was estimated by performing a line fitting on the magnitude of 2D FFT result. Finally, the FM velocity of a bat call was acquired by calculating the slope of the velocity line.

##### Best velocity (BV)

**Figure 7.**
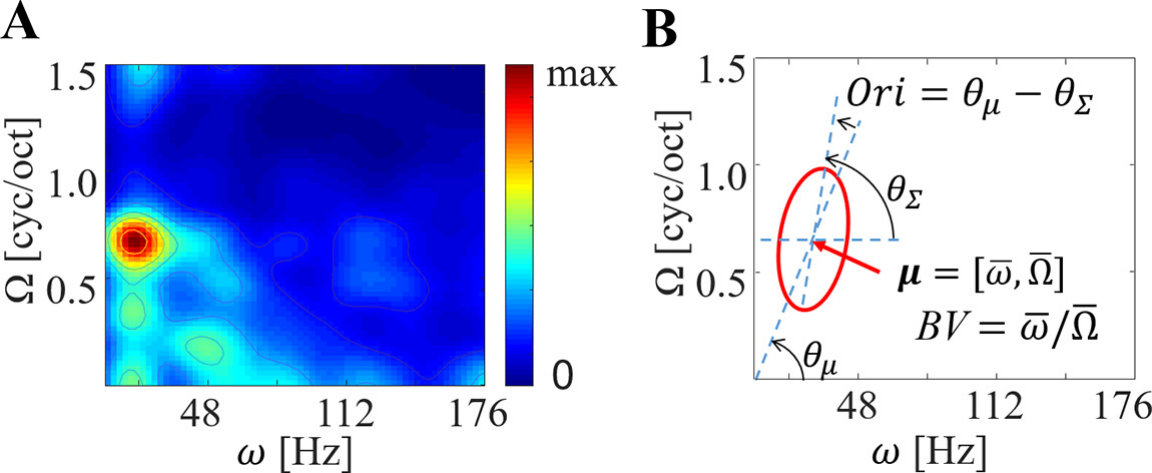
Descriptions for BV and orientation. ***A***, Magnitude plot. ***B***, The result of Gaussian surface fitting. The red ellipse represents Gaussian mean vector *μ* (center) and covariance matrix Σ (rotation), the BV is defined by a slope of Gaussian mean vector and the orientation error is defined by an angle difference between mean vector and covariance rotation.

We defined a BV as the center of mass with respect to response power in a magnitude plot. To estimate the center of mass, we performed a Gaussian surface fitting on the first quadrant of magnitude plot. After normalization as ${\bar{M}}_{e}=M_{e}/[\underset{\text{Ω},\omega }{\sum }M_{e}\Delta \text{Ω}\Delta \omega ]$ where Δω and ΔΩ are, respectively, step size of temporal and spectral modulation rate, the fitting was performed to estimate mean vector and covariance matrix, by minimizing a square mean error function as $Err=\frac{1}{2}\underset{\text{Ω},\omega }{\sum }{\left(\ln\left({\bar{M}}_{e}\right)-ln(G_{\mu ,\text{Σ}})\right)}^{2}$, where $G_{\mu ,\text{Σ}}$ is a Gaussian distribution with mean vector *μ* and covariance matrix Σ. By performing the least square error (LSE) estimator iteratively (Kay, 1993), we derived the Gaussian mean vector and covariance matrix. BV was defined as the slope of the mean vector (Fig. 7).

##### Orientation (Ori)

To characterize velocity selectivity, we defined orientation as the angle between a line connecting the origin to the center of mass and a dominant eigenvector of the Gaussian covariance matrix. Note that the dominant eigenvector indicates the dominant direction of magnitude spread at the center of mass (Fig. 7; Andoni et al., 2007).

##### Inseparability (Ins)

Singular value decomposition (SVD) is applied to each STRF for calculating inseparability (Depireux et al., 2001). This approach decomposes the STRF into a linear combination of rank-1 matrices; in other words, $STRF=\underset{i}{\sum }{\text{λ}}_{i}u_{i}v_{i}^{H}$, where $u_{i}$ and $v_{i}$ are, respectively, left and right eigenvectors (column vector) corresponding to a singular value ${\text{λ}}_{i}$, and *H* means Hermitian transpose (Strang, 2009). Based on this definition, a STRF is called separable if the STRF can be approximated by summation of just a few matrices otherwise it is inseparable. We measured inseparability of a STRF calculated as $Ins=1-\lambda_{1}^{2}/\underset{i}{\sum }\lambda_{i}^{2}$, where $\lambda_{1}\geq \lambda_{2}\geq \lambda_{3}\geq ...$. Note that the inseparability is bounded within the interval [0,1] where the Ins is equal to 0 for separable STRFs otherwise it goes to 1.

##### Direction selectivity index (DSI)

Toinvestigate direction selectivity of STRFs, we compared total power in the first and the second quadrant of the RTF. If a STRF favors downward-moving ripples, total power in the first quadrant of magnitude plot is larger than the other since the first quadrant is composed of responses evoked by downward-sweeping ripples. From this perspective, a DSI was defined as $DSI=(P_{2}-P_{1})/(P_{2}+P_{1})$ where $P_{i}$ is a power in the $i^{th}$ quadrant of RTF, and it is calculated by $P_{i}=\underset{(\text{Ω},\omega )\in Q_{i}}{\sum }|T\left(\text{Ω},\omega \right)|$ where $Q_{i}$ means the $i^{th}$ quadrant. Since the power on each quadrant is a non-negative value, the DSI is bounded within the interval [−1,1] where downward/upward selectivity is represented to negative/positive DSI while 0 represents no selectivity in the direction. DSI for natural vocalizations was derived using the Fourier representation described to derive FM velocity.

##### Best frequency (BF)

To investigate frequency selectivity of STRFs, we defined a BF as the frequency of the maximum peak of absolute STRF, |STRF| over the entire time and frequency spans. BF (spectral peak) of natural calls was computed by finding the peak frequency of the average spectrum.

#### A bootstrap for statistical comparison

We performed a bootstrap analysis to evaluate similarity between distributions of characteristics (e.g., FM velocity, BV) comparing natural calls, IC neurons, and artificial neurons. The procedure selects random 30 samples from natural calls in each iteration with replacement. For IC neurons, random samples from each of the four bats are used in each iteration to maintain a balanced representation across bats. In case of artificial neurons, we trained 10 independent networks (using different initialization procedures) and combined the neurons from each network into a complete set that was then sampled during the bootstrap procedure. For each comparison and each bootstrap repetition, the distance between means was noted. A total of 1000 repetitions were used to generate a distribution of mean distances $d_{\left(\mu ,\text{σ}\right)}$ where *μ* and σ are the mean and standard deviation. The *p* value for accepting null hypothesis was calculated as $p=1-2\overset{\left|\varepsilon \right|}{\underset{0}{\int }}d_{\left(0,\text{σ}\right)}\left(x\right)dx$ where $d_{\left(0,\text{σ}\right)}$ is a zero-mean Gaussian distribution with same variance σ, and *ε* was a real number satisfying $d_{\left(0,\text{σ}\right)}\left(\varepsilon \right)=d_{\left(\mu ,\text{σ}\right)}(\varepsilon )$.

### Natural sound representation with artificial neurons

#### Analysis of response selectivity in artificial neurons

We explored response selectivity to bat calls in biomimetic neurons. To replicate the study performed on IC neurons (Salles et al., 2020), FMB and Echo calls in the sound database were used to measure responses on artificial neurons. Each audio clip was fed into the network after converting to auditory spectrogram (see above, Artificial network front-end processing), then we obtained activation probabilities for 100-nodes as $\sigma (v_{c})$ in Figure 4. We averaged the activation probabilities over the same type of calls, and placed the results for 100-nodes onto a 2D scatter plot. Since the IC neurons are categorized into three groups, FMB selective, Echo selective, and non-selective (Salles et al., 2020), we performed k-means clustering (k = 3) on the principal axis by the principal component analysis (PCA).

#### Social call representation with artificial neurons

We explored bat’s call representation with the biomimetic network. In order to perform stochastic analysis, we made 10-copies for each audio clip in the natural sound database (see above, Social calls for natural sound representation) by a data augmentation based on temporal shift so that 260 audio clips were ready for the response analysis on artificial network. After converting the audio clips for 8 types of bat call to auditory spectrogram (see above, Artificial network front-end processing), the spectrograms were fed into the network to obtain the network’s responses, the $v_{c}$ in Figure 4. Then, we estimated the Gaussian distributions for the responses to each call type, and measured a distance between two distributions by using the Jensen–Shannon divergence (JSD) as JSD(P,Q)=(KLD(P,M)+KLD(Q,M))/2, where *P* and *Q* represent two target distributions, KLD is the Kullback–Leibler divergence (KLD), and M=(P+Q)/2 (Endres and Schindelin, 2003). Unlike the KLD, the JSD is bounded within the interval [0,1] where 0 means that two distributions are equal. Finally, we quantified a discriminability across the classes by averaging JSDs of all cases choosing 2 of 8. In evaluation, we calculated the averaging JSDs with 10-models for each configuration that were trained on different initial values and summarized the mean and standard deviation of the 10 results. Additionally, we explored the noise effect on the sound representation with simulated audios produced by adding Gaussian random noise to each of the 260 audio clips depending on signal-to-noise ratio (SNR).

To compare with neural data, we performed this analysis between FMB and Echo responses. In the same manner, we calculated JSD based on the networks. We adopted neural data used in the previous study (Salles et al., 2020). Among 575 neurons, we chose 351 neurons which were recorded with same version of stimuli, and constructed 351D vector to represent response pattern across the neurons by concatenating the number of spikes on each neuron. Once the vector is projected onto 100D space based on PCA, we estimated Gaussian distributions for FMB and Echo responses. Then, we calculated JSD between the distributions.

## Results

### Database of natural big-brown bat calls

**Figure 8.**
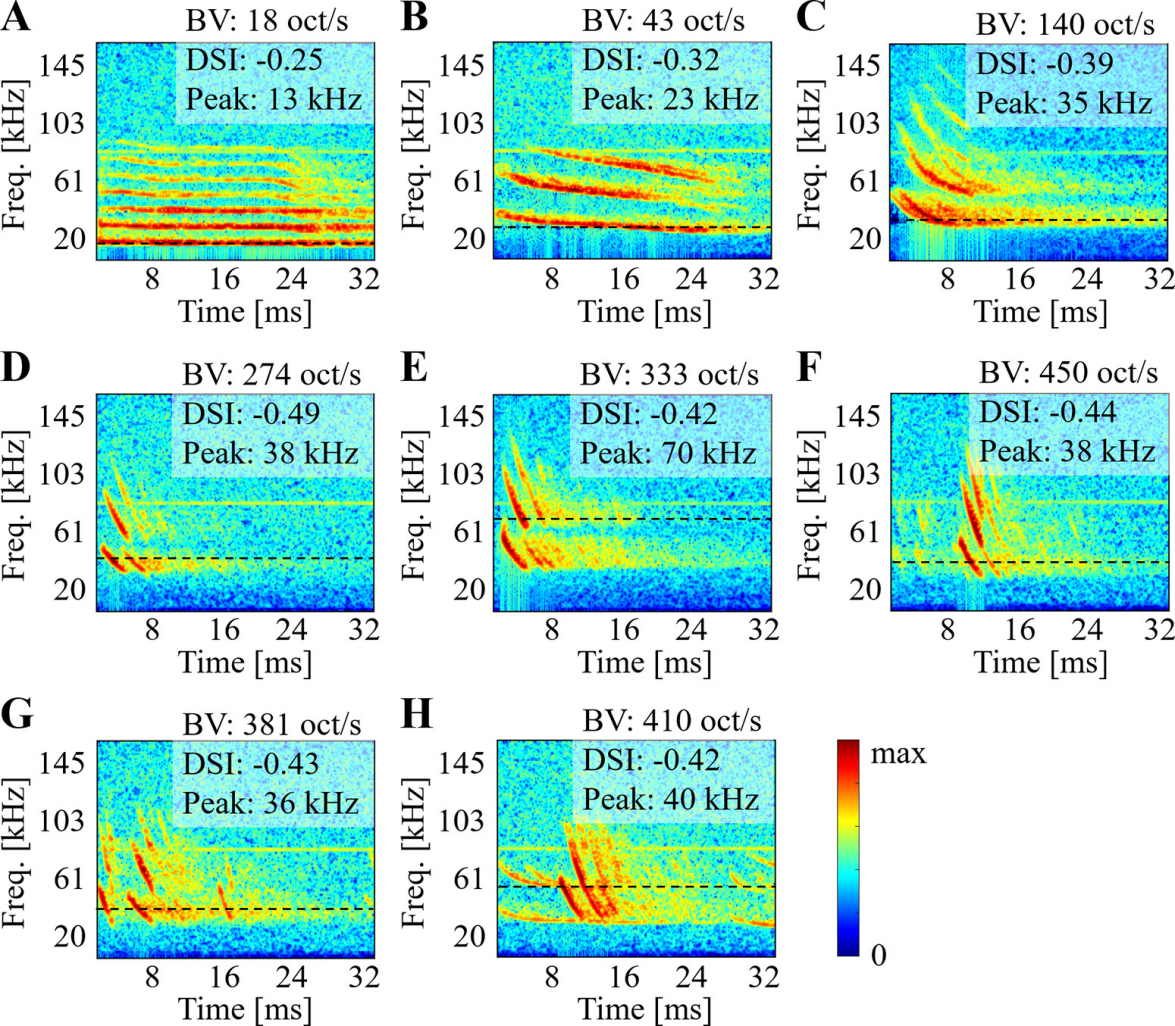
Example spectrograms of several types of calls monophonic cases for social communication (***A–C***), Echo (***D–F***), and polyphonic cases (***G–H***). Note the differences in frequency content, duration, and sweep velocity. Note that peak frequency is represented onto each panel as the black dashed line.

Acoustic recordings of bat calls emitted while socially housed in the laboratory yielded a data set of natural calls containing a wide range of vocalization types. Figure 8 shows the time-frequency representation of several types of vocalizations in the database. The bat vocalizations include isolated (non-overlapping) calls representing communication (Fig. 8*A–C*) or echolocating (Fig. 8*D–F*) sounds as well as overlapping calls from two distinct bats (Fig. 8*G*,*H*). BV values reflect the broad range of FM energies in these social communication calls (BV = 18 oct/s, 43 oct/s and 140 oct/s; Fig. 8*A*,*B*,*C*, respectively). Echo calls show even higher FM energies with shorter signals (BV = 274 oct/s and 333 oct/s; Fig. 8*D*,*E*, respectively). In Figure 8*G*,*H*, we note presence of multiple calls though the statistics derived from that segment are largely influenced by the dominant call (BV = 381 oct/s and 410 oct/s; Fig. 8*G*,*H*, respectively). The natural complexity in the animal’s auditory environment was maintained in this study and no supervised curation of these data set was performed beyond removal of silence segments (see Materials and Methods). We also note presence of ambient background in all recordings as a result of the cage environment and recording setup used to collect these data.

### Auditory characteristics of biological STRFs

**Figure 9.**
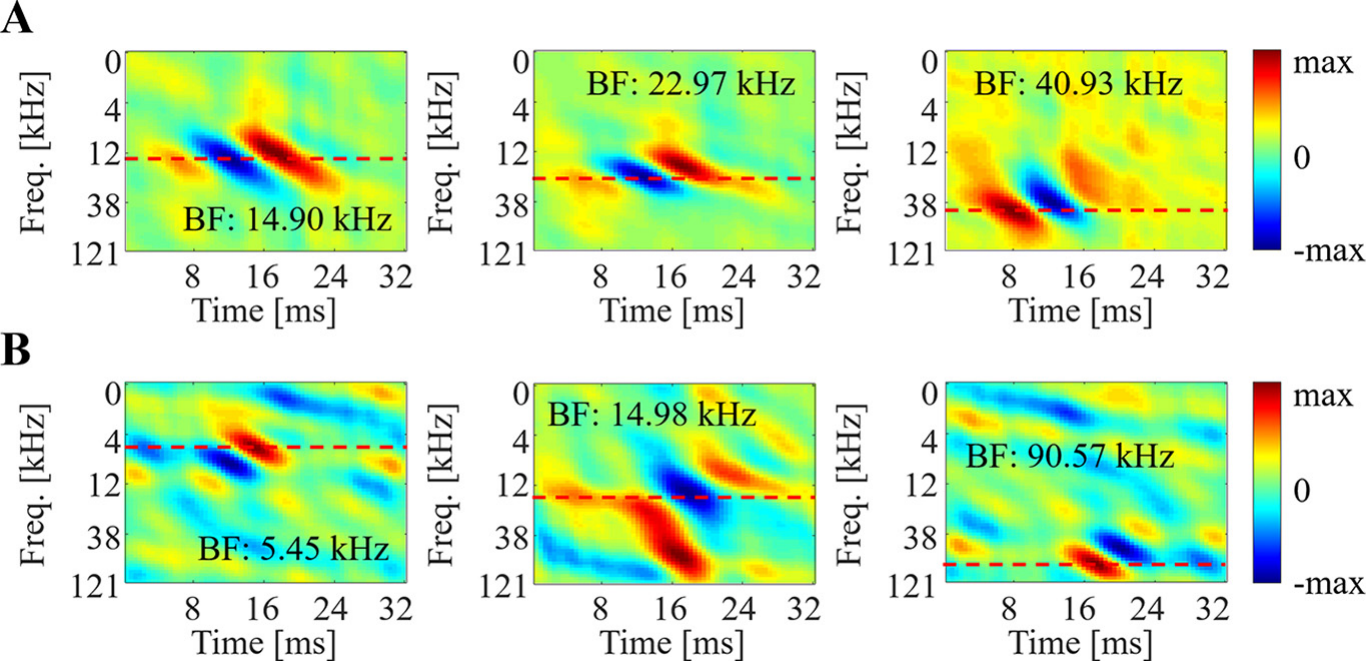
Examples for biological STRF and biomimetic STRF. ***A***, Biological STRFs obtained from bat’s IC neuron. ***B***, Biomimetic STRFs obtained from a triple stacking network with 10% sparsity. Note that red and blue area show excitation and inhibition regions, respectively.

**Figure 10.**
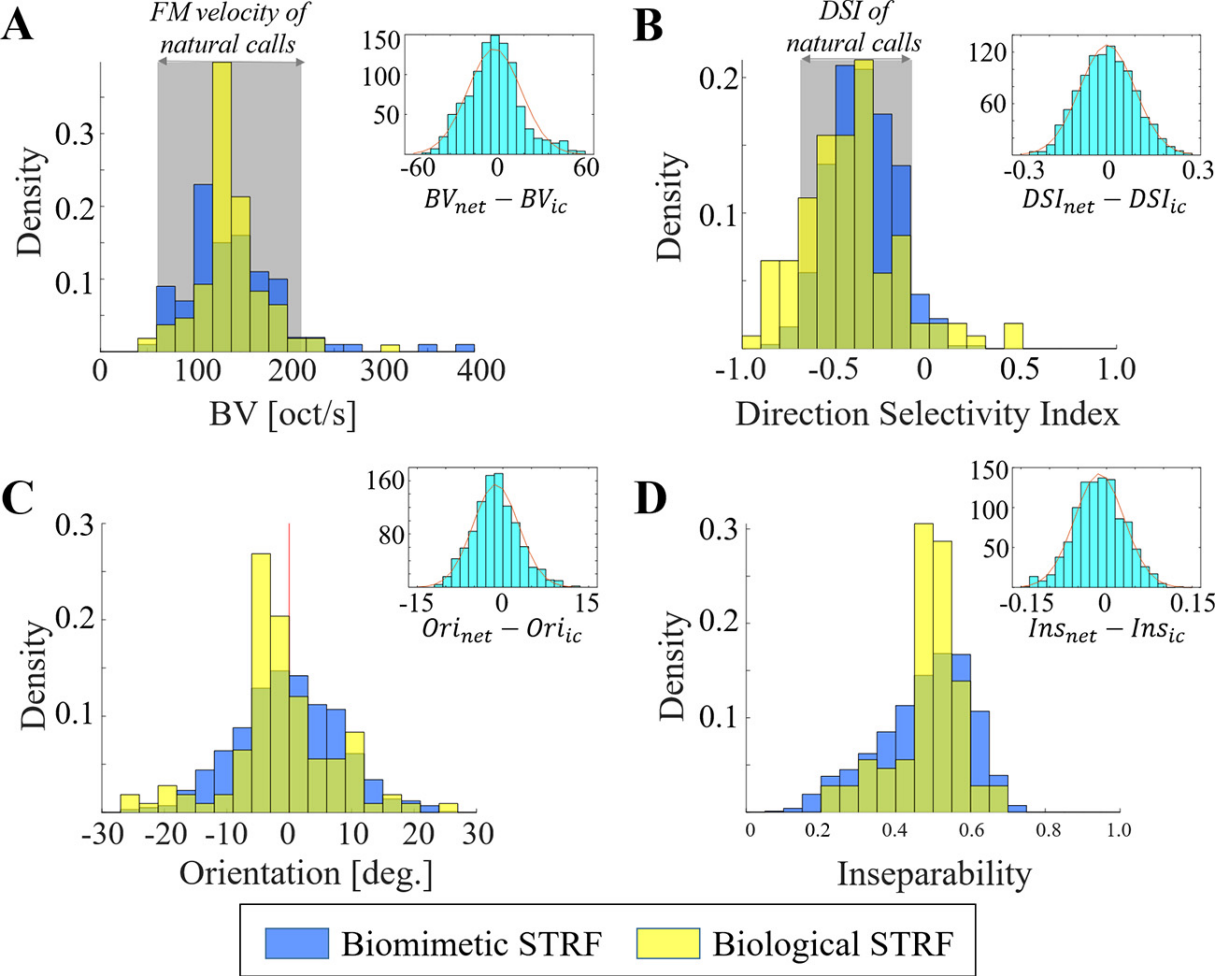
Histogram of biological and biomimetic STRFs according to auditory characteristics. ***A***, BV. ***B***, DSI. ***C***, Orientation (the zero-mean is marked as the red line). ***D***, Inseparability.

To explore auditory characteristics of big brown bat midbrain, we calculated STRFs from neural recordings of IC neurons. Figure 9*A* highlights examples from 6 neurons, revealing a downward sweep selectivity, with excitation and inhibition represented as red and blue areas, respectively. The BF is also shown as red dashed line indicating the maximum peak, positive or negative, of the STRF. We evaluated auditory characteristics across all neural recordings with respect to BV, DSI (direction selectivity), orientation, and inseparability (Fig. 10, yellow histograms). Using a bootstrap procedure, we compared the auditory characteristics of IC neurons to properties of natural calls (Fig. 10, gray background regions for standard deviation). The analysis revealed that the distribution of BVs in IC neurons is statistically equivalent to that of natural calls (*μ* = −1.63, *σ* = 17.13, *p *=* *0.9622; Fig. 10*A*). A match was also observed for direction selectivity *μ* = −0.01, *σ* = 0.03, *p *=* *0.8789; Fig. 10*B*). This result is consistent with the hypothesis that IC neurons have consistent tuning to the statistics of conspecific vocalizations (Andoni et al., 2007). We noted that the majority of IC neurons (93.6%) favored downward sweeps (Fig. 10*B*; Gittelman et al., 2009), while their orientation is centered around 0°. Most IC neurons yield higher than rank-one STRFs (average inseparability index 0.49 ± 0.09).

**Figure 11.**
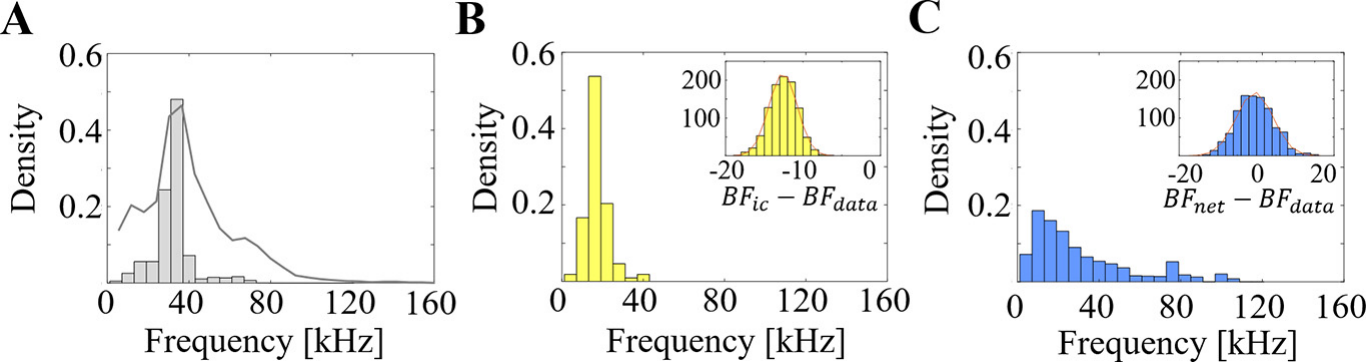
Analysis of BF in dataset, IC neurons, and artificial neurons. ***A***, Histogram of peak frequencies in natural calls, the background gray line represents averaging spectrum envelop of natural calls. ***B***, BFs on IC neurons. ***C***, BFs on artificial neurons.

The distribution of frequency tuning (BF) of IC neurons tended to fall between 10 and 30 kHz. Particularly, BFs of 87% of neurons are below 30 kHz (Fig. 11*B*). In contrast, spectral peaks observed in the vocalization database revealed a higher spectral peak (37.17 ± 5.62) as shown in Figure 11*A*. This profile is likely driven by the strength of the first harmonic component in vocalization which tends to be stronger than other components. As seen from the examples in Figure 8, most vocalizations contain multiple harmonic peaks with higher energy in the first component resulting in a difference between the BF of IC neurons and spectral peaks of the calls database (*μ* = −12.58, *σ* = 1.82, *p *=* *0).

### Auditory characteristics of artificial STRFs

Using natural calls, an artificial network was trained to best represent the statistics of the vocalization. Characteristics of model neurons were analyzed in the same way as biological neurons using STRFs. The distribution of model characteristics is shown in Figure 10, overlaid in blue. Compared with natural calls, model neurons reveal a statistically matching distribution with respect to BV (bootstrap *μ* = −2.62, *σ* = 19.85, *p *=* *0.9473) and DSI (bootstrap *μ* = −0.005, *σ* = 0.01, *p *=* *0.9382). Model neurons also match the spectral peak of natural calls (bootstrap *μ* = −0.49, *σ* = 4.75, *p *=* *0.9592; Fig. 11*C*). These results are not surprising given that the model was trained to mimic the statistics of these calls. Still, the model was not specifically configured to match specific directionality or velocity patterns but rather represent the time-frequency profile of the calls as a whole.

In parallel, the comparison between model and biological neurons reveals remarkable agreement. A bootstrap procedure was performed to compare all auditory characteristics of these STRFs, and results are shown in inset panels in Figure 10. We note that characteristics of biomimetic neurons match the properties of IC neurons including BVs (*μ* = 2.92, *σ* = 16.61, *p *=* *0.9300), DSI (*μ* = 0.01, *σ* = 0.07, *p *=* *0.9358), orientation (*μ* = 1.34, *σ* = 3.26, *p *=* *0.8370), and inseparability (*μ* = −0.02, *σ* = 0.04, *p *=* *0.8079). The BFs of artificial neurons are statistically different from IC neurons (bootstrap *μ* = 12.10, *σ* = 4.44, *p *=* *0.1731), although there is substantial overlap at the range of 0–40 kHz. The BFs of artificial neurons are more broadly distributed over the entire frequency range with ∼14% of artificial neurons having high BF (above 60 kHz; Ferragamo et al., 1998).

### Architecture of the biomimetic network

**Figure 12.**
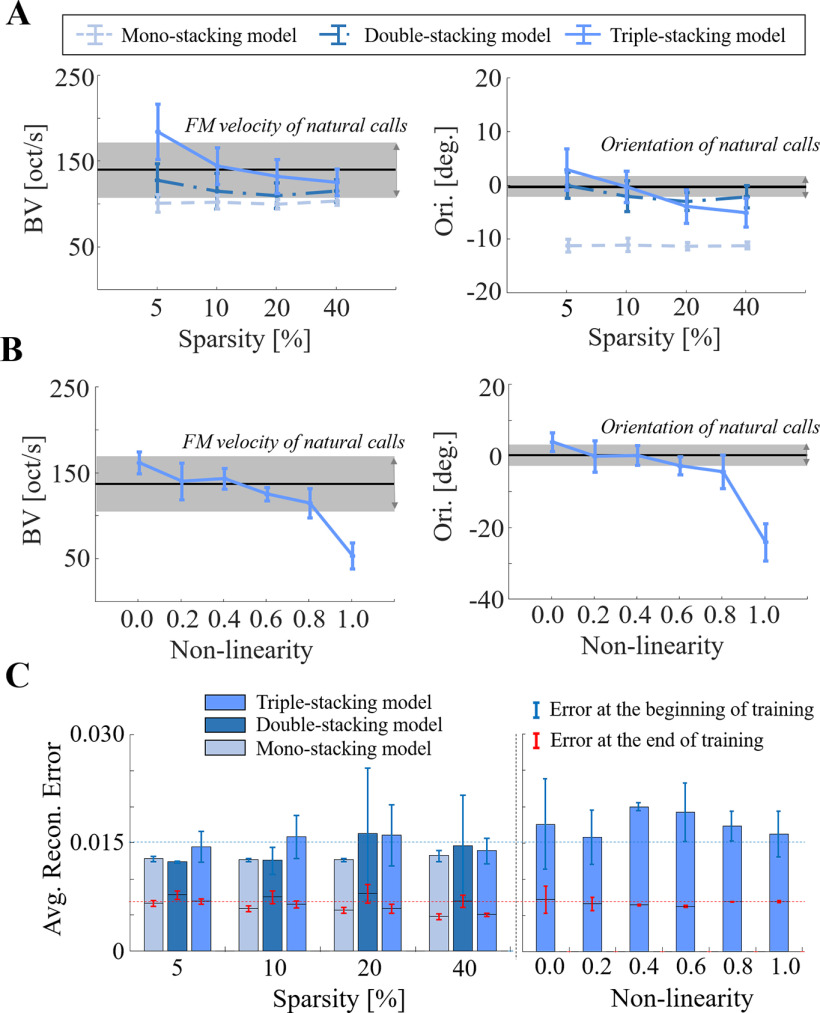
BV and orientation of biomimetic STRFs depending on network’s configuration (***A***) for the number of stacking modules and sparsity (0.2 lReLu), (***B***) for nonlinearity (triple stacking model with 10% sparsity), and (***C***) average reconstruction error over the 10 networks for each parameter set; blue and red dashed lines are mean of the errors for all configurations at the beginning of training and the end of the training, respectively.

While results reported so far focus on the “best” biomimetic network, we also investigated how changing the architecture of the model affects the tuning parameters of artificial neurons. We systematically varied the model in terms of structural complexity (the number of stacking blocks), sparsity of the latent space and non-linearity of the activation function. Figure 12*A* shows the mean and standard deviation of characteristics of model neurons across 10-network validations for each pair of complexity and sparsity (α=0.2). The mean FM velocities and orientation in the natural calls database are represented by a black line on each panel; while the gray regions represent 95% confidence intervals for each mean. The results show that a very shallow model (mono-stacking) results in a greatly biased negative orientation, as well slower BV estimates. By increasing the model depth, there is an increased match between the model’s spectro-temporal configuration (represented by BV and orientation) and that of natural statistics. Furthermore, extremely low or high sparsity values also result in over or underestimating statistics of natural calls; with 10% sparsity results in a great match with average statistics of the natural calls.

Using the triple stacking network with 10% sparsity, we investigated the effect of the model non-linearity on the same auditory characteristics of model neurons (Fig. 12*B*). Setting the non-linearity parameter to 1.0 results in a fully linear processing which clearly produces in a mismatch between the model and call characteristics. By increasing the degree of non-linearity (decreasing α), we note a closer match between the two.

It should be noted that across all the different configurations of the model, all architectures were able to converge (i.e., minimize the reconstruction error between the spectrogram of a given call sound and its reconstruction using the model’s latent space). Figure 12*C* shows the average reconstruction error over the 10 models for each parameter set. While all models successfully converge to reconstruct natural calls and encode statistics of in the database, only a few configurations result in a reasonable match to the spectro-temporal characteristics of model neurons. As a matter of fact, the model was not constrained to match these properties in its latent space; it is merely trained to represent the call spectrograms as faithfully as possible. This requirement has multiple plausible solutions, and only certain configurations result in a close match with velocity and orientation characteristics of natural calls.

### Natural call representation with the biomimetic network

**Figure 13.**
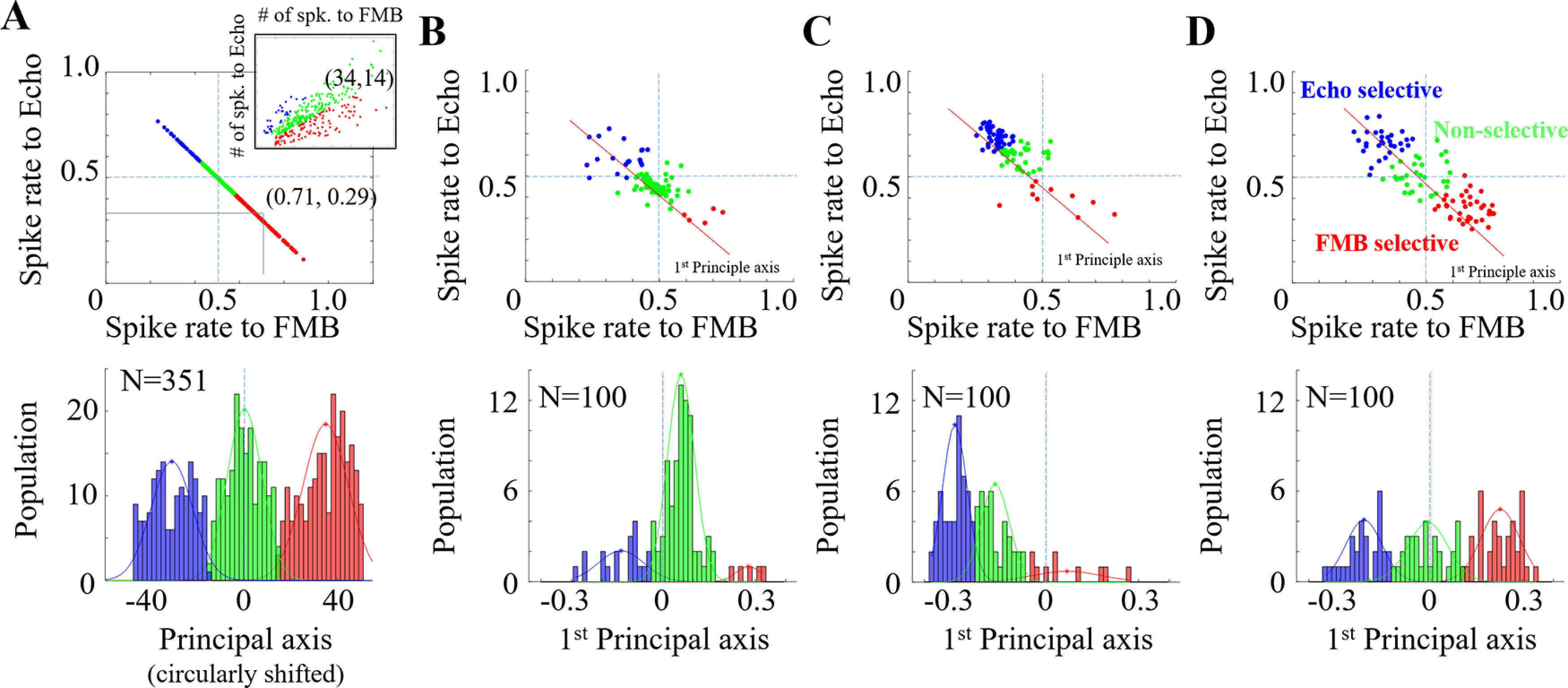
Selectivity to FMB versus Echo call for (***A***) IC neurons (Salles et al., 2020), spike frequency was calculated by dividing the number of spikes by total number of spikes starting 5 ms after stimulus onset. The horizontal axis on the bottom was circularly shifted with zero-centered non-selective neurons. (***B***) Mono-stacking model. ***C***, Double-stacking model. ***D***, Triple-stacking model.

So far, the results suggest that a deep nonlinear architecture with high sparsity to achieve an optimal representation of the statistics of natural bat vocalizations is capable to replicate auditory characteristics of the bat’s midbrain. We next examined the implications of this mapping to facilitate discrimination of the large variety in the call repertoire. A study revealed that tuning characteristics of bat IC neurons differentially encode different sound categories in the bat vocalizations, specifically Echo calls and food-claiming FMB social calls (Salles et al., 2020). We examined whether the artificial network, trained simply to emulate natural statistics in the bat repertoire (without knowledge of different sound classes) also yields distinct activations of these different groups. Figure 13*A*, top, replicates the response selectivity of biological IC neurons, showing a scatter plot of average activation probabilities for each neuron in response to FMB calls (*x*-axis) versus Echo calls (*y*-axis), projected on the principal axis by PCA. The figure inset shows the original neural responses before data projection. Figure 13*B–D* depict a similar analysis of call selectivity for the mono, double and triple artificial network, respectively. Note that each panel from *B* to *D* was produced by one network of 10-models for example. The top panels show a scatter. Across the three network configurations, we note that the mono stacking model induces mostly non-selective activation across its neural population (Fig. 13*B*), while the double stacking model yields biased responses in favor of Echo calls (Fig. 13*C*). The triple stacking model reveals a more balanced activation from Echo and FMB social call types (Fig. 13*D*) that closely matches biological selectivity.

**Figure 14.**
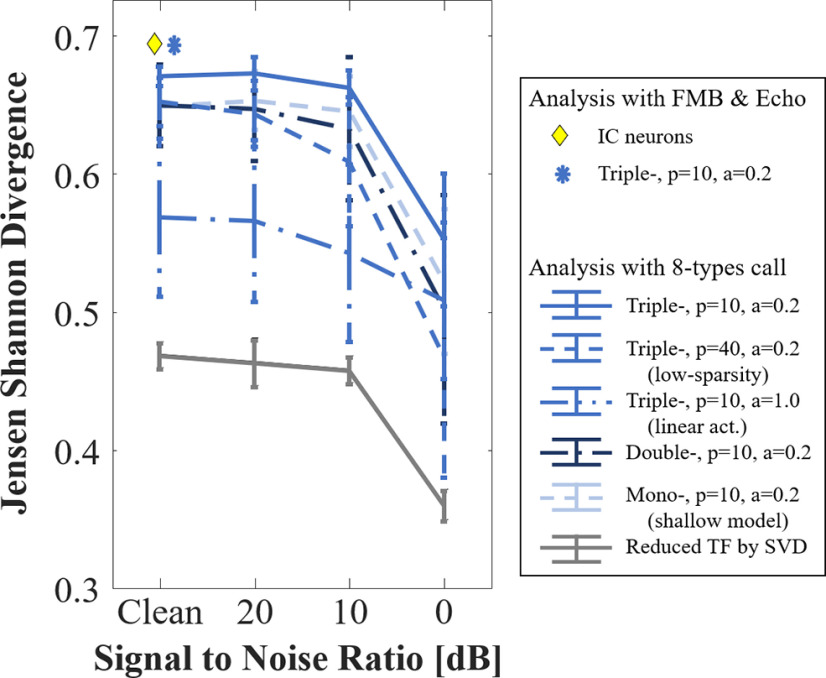
Natural sound representation by biomimetic network in different SNR conditions.

We extend the analysis of call selectivity in the artificial network to other classes of calls in the bat repertoire. We evaluated discriminability across eight types of calls using the JSDs (Endres and Schindelin, 2003). Figure 14 shows the results for various network depths, linearity and sparsity for the calls in the database (clean) as well as with additional simulated additive noise with decreasing SNRs. The triple stacking model (with high sparsity and nonlinear activation) produces the most discriminable responses, as well as more robust discrimination even in presence of noise. Shallower architectures are clearly affected by presence of resulting in reduced discriminability. Linear activations and low sparsity appear to also affect discriminability and robustness to noise albeit not at the same rate. These results suggest that the optimal representation of call statistics likely plays a role in facilitating the identification of different sound classes even in presence of noise. Similarly, a study with guinea pigs has shown the robust discrimination in the responses to communication sounds (Souffi et al., 2020). Such hypothesis aligns with earlier reports (Chechik et al., 2006) but remains to be validated in the IC of the big brown bat. As reference, we computed JSD for the two echo and FMB call classes (Fig. 14) for both artificial and biological neurons. Both measures reveal a close agreement and high discriminability that far surpasses selectivity from shallower architectures.

## Discussion

### The biomimetic artificial network provides a nonlinear response model of neural selectivity

To examine the tuning of auditory neurons, each cell can be considered as a system with a mapping function *F* that represents a relation between stimulus *s* and neural response *r*, i.e., r=F(s). While characterizing the full system function may be theoretically or experimentally nearly impossible, linearized models using STRF are often used to build a computational response model as $r\left(t\right)=\int^{}s\left(t,f\right)*h\left(t,f\right)df$, where *t* and *f* is, respectively, time and frequency index, s(t,f) is spectro-temporal representation for a stimulus, * is a convolution operator, and h(t,f) represents a STRFs (Depireux et al., 2001; Fritz et al., 2003; Machens et al., 2004; Elhilali et al., 2013). This model is often applied with reasonable success to predict neural responses to other sound classes including conspecific vocalizations or other natural sounds. Although the linear model is a reasonable approximation for mimicking neural responses in the brain, it is limited in its ability to inform nonlinear transformations that are usually observed in between stimulus and response (Theunissen et al., 2000; Escabi and Schreiner, 2002). One of the main advantages of including nonlinear activations in a feed-forward propagation in the proposed neural network is that it implicitly incorporates the effects of these nonlinear mappings in the propagation of activity throughout the network. Still, we are able to evaluate the linearized portion of the response (via STRFs of artificial neurons) without explicitly incorporating the nonlinear terms in the STRF model itself. This black-box approach to incorporate complexities of neural mapping via deep neural networks opens the possibility to more intricate readouts of the representation of artificial networks. We anticipate that such biomimetic artificial network can be used to build a system mimicking the bat’s ability for object shape recognition using its bio-sonar.

### Midbrain responses are optimized to represent the statistics of natural calls in a bat’s soundscape

In this study, we explored the hypothesis that the bat’s IC neurons are tuned to represent the FM velocity and spectro-temporal structure of conspecific vocalizations. Evidence in support of this Sender-Receiver matching has been previously reported in the pallid bat (Fuzessery, et al., 2006) and Mexican free-tailed bat (Andoni et al., 2007) as well as other species such as zebra finches (Woolley et al., 2005; also see Woolley and Portfors, 2013). Here, we report similar findings in the big brown bat, and establish a close correspondence between acoustic characteristics of natural calls and tuning of STRFs of IC neurons of the big brown bat. Going beyond this relationship, an artificial network trained independently on these natural calls reveals tuning properties that not only conform with spectro-temporal features of the calls (which they were trained on), but also unveils IC-like tuning structure and complexity (e.g., separability) that the model was not specifically trained on (Fig. 10). This result hints that the midbrain architecture gives rise to tuning configurations that leverage the spectro-temporal richness of the bat’s repertoire to not only represent these features with high fidelity but also enable selective responses to discriminate between classes of natural calls.

The artificial network used in the current study shows that the neural encoding of an incoming stimulus gives rise to a response across neural populations that enables it to faithfully reconstruct this stimulus, revealing a high-fidelity mapping without loss of information. While not explicitly happening in the brain, this stimulus reconstruction from the internal latent space is the basis for training the artificial network which yields emergent tuning that matches the biology. It is important to note that tuning properties of artificial neurons were derived using moving ripples which invoke the principle of signal decomposition by separating each conspecific call into a sum of ripples with different orientations, rates and phases, in line with the Fourier theory of signal representation. While the network was never trained on these ripples, its response to each ripple spectral motion pattern both in terms of magnitude and phase (both needed for STRF reconstruction) suggest a quantitative correspondence with the downward-sweeping signals that are prominent in the bat repertoire. It is also important to note that not all known coding properties of the bat midbrain are represented in STRFs (Brimijoin and O’Neill, 2010) and that future steps to test time varying response properties (such as an adaptation) would further validate the ability of this network to replicate the biology of the bat IC (Lesica and Grothe, 2008; Rabinowitz et al., 2013; Lohse et al., 2020).

### A deep architecture with sparsity is best suited to model the statistics of natural calls

Varying the architecture of the network led to different latent spaces to represent the characteristics of the database of natural calls. Specifically, changing the complexity of the network (in terms of depth), sparsity and nonlinearity converged on different solutions for representing conspecific sounds. Under all configurations, the networks were able to reconstruct the input spectrogram with minimal error indicating that its latent space is sufficiently informative about the statistics in the training database (Fig. 12*C*). Nonetheless, only a specific configuration with high sparsity, nonlinearity and sufficient depth is able to replicate biological tuning properties, giving insights into coding principles underlying configuration of IC networks probed in this study. Naturally, while this investigation cannot rule out other configurations that would also reveal a strong match to biology, it can eliminate parameters that converge on solutions that are far from the biology (e.g., shallow networks, linear models). It is worth noting that we were unable to train a quadruple stacking network to represent statistics in the database so we are unable to comment on the extent to which an even deeper network may correlate with biological tuning. The output of a fourth block could be missing spectro-temporal features because of overcompression. This is an issue that could explored using large input patches or modifying the pooling step.

### Tuning to conspecific natural sounds may underlie selective and robust encoding of auditory objects

We note that directional selectivity to FM sweeps in biological and artificial neurons, results in high discriminability between different classes of calls. Specifically, these results support the notion that by having neural sub-population tuned to different subsets of spectro-temporal statistics, the network is able to encode and differentially respond to different vocalizations and social or Echo calls. This discriminability is enhanced in the triple sparse and nonlinear network that best matches biological tuning and much reduced in other network configurations despite the fact that these other models were also successfully trained to represent the same natural statistics in the bat call repertoire. This variability may stem from correlated behavior across the neural population which was previously shown to play an important role in enhanced discriminability of vocalizations in the auditory midbrain (Schneider and Woolley, 2010). This encoding selectivity remains fairly stable in presence of stationary ambient noise suggesting that the high dimensional mapping encoding incoming natural calls results in a noise invariant representation that is believed to start emerging at the level of the IC and further strengthen in auditory cortex (see Willmore et al., 2014).

## Synthesis

Reviewing Editor: Christine Portfors, Washington State University Decisions are customarily a result of the Reviewing Editor and the peer reviewers coming together and discussing their recommendations until a consensus is reached. When revisions are invited, a fact-based synthesis statement explaining their decision and outlining what is needed to prepare a revision will be listed below.

Thank you for so thoroughly revising the manuscript based on previous reviewers' comments.
